# Myoblast Migration and Directional Persistence Affected by Syndecan-4-Mediated Tiam-1 Expression and Distribution

**DOI:** 10.3390/ijms21030823

**Published:** 2020-01-27

**Authors:** Daniel Becsky, Szuzina Gyulai-Nagy, Arpad Balind, Peter Horvath, Laszlo Dux, Aniko Keller-Pinter

**Affiliations:** 1 Department of Biochemistry, Faculty of Medicine, University of Szeged, H-6720 Szeged, Hungary; becsky.daniel@med.u-szeged.hu (D.B.); szuzina1997@gmail.com (S.G.-N.); dux.laszlo@med.u-szeged.hu (L.D.); 2 Institute of Biochemistry, Biological Research Centre, Hungarian Academy of Sciences, H-6726 Szeged, Hungary; balind.arpad@brc.hu (A.B.); horvath.peter@brc.hu (P.H.)

**Keywords:** myoblast, cell migration, Tiam1, syndecan-4, proteoglycan, Rac1, NSC23766, directional persistence, muscle regeneration, skeletal muscle

## Abstract

Skeletal muscle is constantly renewed in response to injury, exercise, or muscle diseases. Muscle stem cells, also known as satellite cells, are stimulated by local damage to proliferate extensively and form myoblasts that then migrate, differentiate, and fuse to form muscle fibers. The transmembrane heparan sulfate proteoglycan syndecan-4 plays multiple roles in signal transduction processes, such as regulating the activity of the small GTPase Rac1 (Ras-related C3 botulinum toxin substrate 1) by binding and inhibiting the activity of Tiam1 (T-lymphoma invasion and metastasis-1), a guanine nucleotide exchange factor for Rac1. The Rac1-mediated actin remodeling is required for cell migration. Syndecan-4 knockout mice cannot regenerate injured muscle; however, the detailed underlying mechanism is unknown. Here, we demonstrate that shRNA-mediated knockdown of syndecan-4 decreases the random migration of mouse myoblasts during live-cell microscopy. Treatment with the Rac1 inhibitor NSC23766 did not restore the migration capacity of syndecan-4 silenced cells; in fact, it was further reduced. Syndecan-4 knockdown decreased the directional persistence of migration, abrogated the polarized, asymmetric distribution of Tiam1, and reduced the total Tiam1 level of the cells. Syndecan-4 affects myoblast migration via its role in expression and localization of Tiam1; this finding may facilitate greater understanding of the essential role of syndecan-4 in the development and regeneration of skeletal muscle.

## 1. Introduction

Skeletal muscle is a highly dynamic tissue that can regenerate successfully following injury, and it can change in size in response to exercise, aging, or diseases (e.g., cancer cachexia, immobilization, muscular dystrophy). The skeletal muscle stem cells, also known as satellite cells, which are localized between the muscle fiber and basal lamina, are responsible for the plasticity, maintenance, and regeneration of skeletal muscle [1,2]. The satellite cells are mitotically and physiologically quiescent in healthy muscle, but they are stimulated by local damage to form myoblasts that subsequently proliferate, migrate, differentiate, and fuse to form multinucleated muscle fibers.

Cell migration is essential for establishing and maintaining the proper organization of multicellular organisms [3]. Cell movement is a complex process that plays an important role in various physiological processes including regeneration, wound healing, angiogenesis, embryonic development, and immune cell responses, in addition to tumor progression and metastasis formation. One of the most important conditions for cell movement is the polarization of the cell, in terms of morphology, with the formation of a leading edge at the front and a retracting tail, and the correct positioning of the cellular components [4]. An actin-rich protrusion, called the lamellipodium, must evolve toward migration, thereby creating asymmetry in the cell, with distinct front-rear polarity [5]. Polarization is also defined by the positioning of the cell nucleus and the reorientation of the Golgi network and microtubule organizing center toward the leading edge [6,7]. The Rho family of small GTPases, including Rac1 (Ras-related C3 botulinum toxin substrate 1), Cdc42, and RhoA, plays a fundamental role in the development and maintenance of this front-rear polarity [8].

The Rho GTPases act as molecular switches cycling between an inactive GDP-bound and an active GTP-bound form to bind and activate downstream effector proteins, thereby affecting various signaling pathways [9]. The two-state cycle is regulated by three sets of proteins: the guanine nucleotide exchange factors (GEFs), which catalyze the exchange of GDP for GTP, and GTPase-activating proteins (GAPs), which increase intrinsic GTP hydrolysis, are responsible for Rho GTPases switching between their active and inactive forms, respectively. Moreover, switching between GDP- and GTP-bound states may involve cytosol-membrane translocation, as guanine dissociation inhibitors (GDIs) prevent Rho GTPases from membrane-targeting and activation [10]. 

Tiam1 (T-lymphoma invasion and metastasis-1) has been identified as a GEF, acting as a specific activator of Rac1 [11], and it plays a key role in pivotal biological processes including cell migration [8] and cell polarization [11]. The Par (partitioning defective) polarity complex, comprising Par3, Par6 and atypical PKC, plays a key role in the development and maintenance of cell polarity [8]. Furthermore, Tiam1, along with the Par polarity complex, stimulates persistent migration by stabilizing the anterior-posterior polarization of migrating cells [12]. Par3 interacts with Tiam1, leading to localized Rac1 activation, creating a gradient of Rac1 and RhoA GTPases in migrating cells: the former is concentrated at the leading edge, and the latter is in the rear of the cell [13]. As Tiam1-mediated Rac1 signaling is required for establishing and maintaining cell polarity [14], impaired Tiam1 signaling inhibits the formation of front-rear polarization in migrating cells thereby inhibiting persistent migration [12].

Syndecan-4 (SDC4) is a ubiquitously expressed transmembrane, heparan sulfate proteoglycan that is a major cell surface marker of satellite cells [15]. Syndecan-4 connects the extracellular matrix to the cytoskeleton, allowing the interaction between the cell and extracellular matrix components, cytokines, or growth factors [16]. The cytoplasmic domain contains one variable and two conserved regions, binding to ezrin, radixin, and moesin proteins, in addition to Src kinase, α-actinin, or protein kinase C α (PKCα) [17,18,19], and regulating intracellular Ca^2+^ concentration [20] or Rac1 activity [21,22]. Syndecan-4 binds and inhibits Tiam1, modulating the activity of Rac1 GTPase [22]. The cytoplasmic domain of syndecan-4 contains a type II PDZ binding site, and Tiam1 exhibit a PDZ domain. Both phosphorylation of the cytoplasmic serine residue of syndecan-4 and the PDZ domain binding of Tiam1 are involved in regulation of the GEF activity of Tiam1, thereby regulating Rac1-GTP level [22]. 

Syndecan-4 knockout mice cannot regenerate injured skeletal muscle [23], and the precise mechanism underlying this phenomenon is unclear. Given the role of syndecan-4 in the regulation of Rac1 GTPase and the importance of cell migration during muscle regeneration, the aim of our study was to investigate the role of Tiam1-mediated Rac1 activation in syndecan-4-dependent myoblast migration. We have demonstrated that syndecan-4 knockdown decreased migration of myoblasts, accompanied by reduced global Tiam1 expression. Moreover, it abrogated the asymmetric, polarized distribution of Tiam1 and reduced directional persistence of the movement. 

## 2. Results

### 2.1. Syndecan-4 Silencing Reduces Myoblast Migration in a Random Migration Assay

We verified the effect of syndecan-4 (SDC4) silencing by qPCR technique and Western blotting in C2C12 mouse myoblast cell lines transfected stably with plasmids expressing shRNA specific for syndecan-4 (shSDC4#1 and shSDC4#2), and we also tested the effect of the scrambled sequence [24]. The syndecan-4 expression was significantly decreased in both shSDC4#1 and shSDC4#2 cell lines, with the reduction being less in the latter; whereas the scrambled sequence did not affect the syndecan-4 level [24]. 

**Figure 1:**
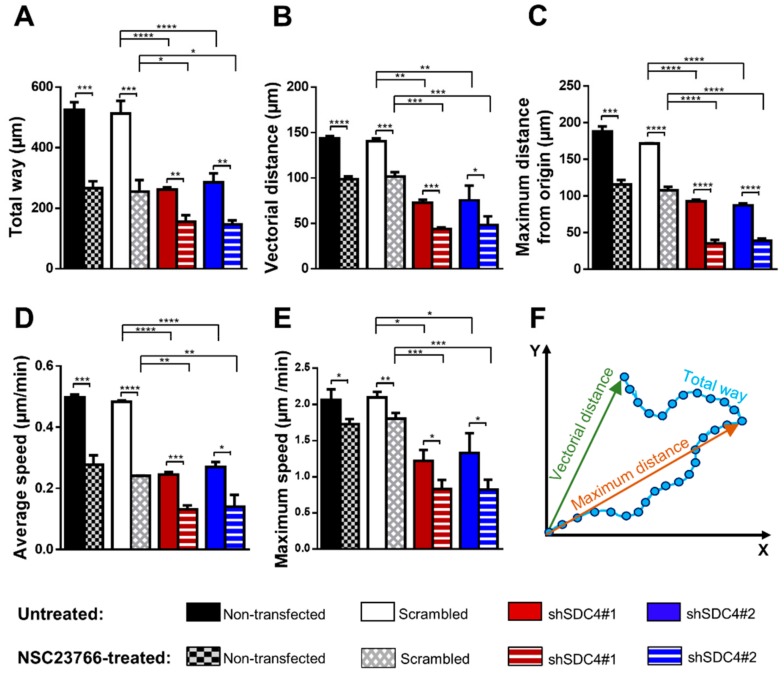
The role of syndecan-4 and Rac1 (Ras-related C3 botulinum toxin substrate 1) in random, two-dimensional migration of myoblasts. The total length of movement (**A**), the vectorial distance (real displacement of the cells) (**B**), the maximum distance from the starting point (**C**), the average cell speed (**D**), and the maximum speed (**E**) of the cells are depicted in non-transfected, scrambled, and syndecan-4 silenced (shSDC4#1, shSDC4#2) C2C12 myoblast cells either without or with NSC23766 (Rac1 inhibitor) treatment. Schematic representation of the total path, vectorial distance, and maximum distance of the movement (**F**). The total duration of live cell microscopy: 18 h, frame rate: 3/1 h; *n* = 4 independent experiments; 62–114 cells/cell line; and 6–8 fields of view/experiment. Data are reported means of the independent experiments + SEM; **** *p* < 0.0001; *** *p* < 0.001; ** *p* < 0.01; * *p* < 0.05.

Initially, the effect of syndecan-4 silencing was examined in a random migration assay for 18 h. Representative time-lapse videos are included as Supplementary Materials Videos S1–S4. Syndecan-4 knockdown significantly reduced the total path of migration, both the vectorial distance (i.e. real shift of the cells) and the maximum distance from the origin were decreased; furthermore, the average and maximum speed were also reduced (Figure 1A–E). No significant difference was observed between the non-transfected and scrambled cells. 

**Figure 2:**
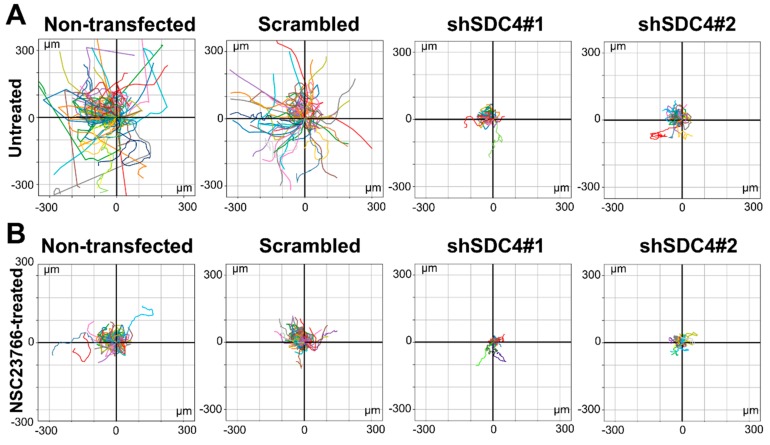
Representative wind-rose plots depict the total path of the cells. The trajectories were shifted to a common origin. Each colored line represents the total path of a single untreated myoblast either without (**A**) or with NSC23766 treatment (**B**) in the different cell lines. Total duration of live cell microscopy: 18 h.

Next, we transposed the migratory tracks (total paths) of the individual cells to a common origin to generate the static wind rose plots depicted in Figure 2. The representative wind rose plots depict the migratory tracks of cells based on the position of the x and y coordinates of the movement (Figure 2). The smaller diameters of the wind rose plots in both shSDC4#1 and shSDC4#2 lines indicate the reduced motility of these cells (Figure 2A). 

### 2.2. Inhibition of Rac1 Does Not Restore the Defective Migratory Phenotype of Syndecan-4 Knockdown Cells

Syndecan-4 knockout causes a steady increase in the levels of activated Rac1-GTPase [21,25,26,27,28]. The aim of our next experiment was to study how Rac1 inhibition affects migration, and whether it can improve the decreased migration of syndecan-4 silenced cells. The activity of Rac1 was specifically inhibited by NSC23766 treatment [29]. Cell migration was examined for 18 h during a random migration assay. Representative time-lapse videos (Supplementary Materials Videos S5–S8) show the decreased motility of the cells following NSC23766 treatment. During this analysis, the specific inhibition of Rac1 GTPase did not ameliorate the migration defect due to syndecan-4 knockdown. Interestingly, Rac1 inhibition caused further significant reduction in all examined parameters, including the total path of the cells, vectorial and maximum displacement, and average and maximum speed values in all cell lines (Figure 1). 

The representative wind rose plots depicting the migratory tracks of the individual cells show the decreased motility of all cell lines upon treatment with the Rac1 inhibitor NSC23766 (Figure 2B). The representative plots clearly show the result of the combined effect of syndecan-4 silencing and Rac1 inhibition. Notably, the migratory parameters of syndecan-4 silenced cell lines further decreased following NSC23766 treatment. 

### 2.3. Syndecan-4 Knockdown Affects the Directional Persistence of Migration

**Figure 3:**
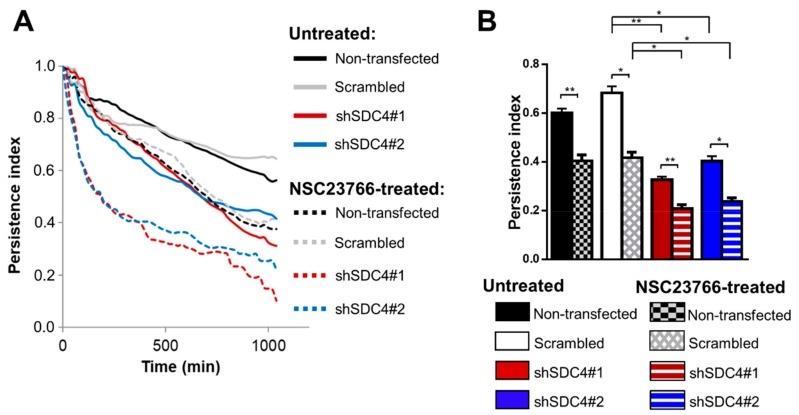
Directional persistence in cell migration. (**A**) Average persistence index (vectorial distance/total path ratio) over elapsed time indifferent cell lines. (**B**) Effect of Syndecan-4 (SDC4) silencing and/or Rac1 inhibition (NSC23766 treatment, 50 µM) on persistence index after 18 h. *n* = 4 independent experiments, 62–114 cells/cell line, 6–8 fields of view/experiment, Data are reported as means of the independent experiments + SEM; * *p* < 0.05; ** *p* < 0.01.

The effectiveness of cell migration depends on two essential features: cell-speed and directional persistence. At the cellular level, directional persistence depends on the tenacity of lamellipodial protrusions and the stability of the trailing edge [27,28]. To ascertain the stability of the orientation of the cell migration, we also calculated the persistence index [29] in the case of the control and the syndecan-4 knockdown cell lines. The values of the persistence index over the timescale represent the time-dependency of the directional persistence showing that these differences can be constantly observed by comparing the cell lines (Figure 3A). 

The results show that silencing of syndecan-4 significantly decreases the persistence index of myoblasts measured after 18 h movement. There was no significant difference between the non-transfected and scrambled cell lines. NSC23766 treatment of the cells further reduced persistence index in both syndecan-4 knockdown cell lines (Figure 3B). Interestingly, the persistence index of the untreated syndecan-4 knockdown cells was similar to the NSC23766-treated control lines; suggesting that neither high nor low activity of Rac1 favors directional persistence of the migration. 

### 2.4. Syndecan-4 Affects Tiam1 Expression and Localization

**Figure 4:**
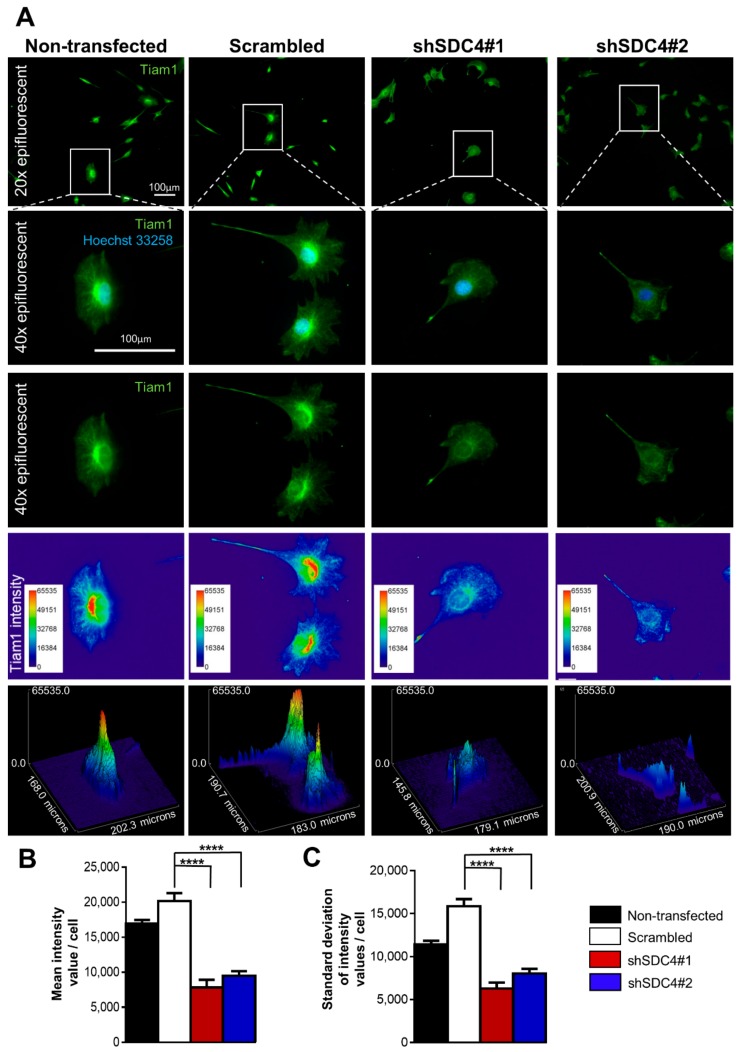
Tiam1 (T-lymphoma invasion and metastasis-1) expression of the different cell lines. (**A**) Representative images show Tiam1 (green) distribution. The non-transfected and scrambled cells exhibit an asymmetric, polarized Tiam1 arrangement, which is absent due to SDC4 silencing. Nuclei are blue (Hoechst 33258). Representative pseudocolor images (2D and 3D) depict Tiam1 signal intensity. The color was assigned to each pixel based on the pixel intensity value according to the calibration bar (shown on left-hand sides of the images). The mean intensity values in the individual cells are quantified in (**B**), and the average standard deviation values of the Tiam1 intensities are depicted in (**C**); *n* = 4–20 cells/cell line were analyzed; data are reported as mean+ SEM; **** *p* < 0.0001.

As the inhibition of Rac1 activity reduced migration ability (in both control and syndecan-4 silenced cell lines), we explored whether we could identify alteration in either distribution or expression of Tiam1. We found an asymmetrical Tiam1 distribution with an increased intensity towards the leading edge in the non-transfected and scrambled cell lines, whereas this peak in the Tiam1-intensity was absent in the syndecan-4 silenced cells (Figure 4A). The representative 2D and 3D pseudocolor images provide better visualization of this altered Tiam1 distribution: the high-intensity red area depicted an asymmetric Tiam1 enrichment that is mainly adjacent to the nucleus, and homogenous cytoplasmic blue-green intensity was observed in both syndecan-4 silenced cell lines. These quantified results confirm the pervious observations, as the mean intensity values in the individual cells are significantly decreased following syndecan-4 knockdown (Figure 4B), indicative of reduced Tiam1 expression in these cells. To measure the variation in the pixel intensities, the standard deviation of Tiam1 intensity values within each cell were quantified. The standard deviation of intensity values in both syndecan-4 silenced cell lines were significantly lower. The pixel intensity values were closer to the mean intensity, whereas they are spread out over a wider range in the control cells (Figure 4C). 

## 3. Discussion

Tiam1 plays an essential role in pivotal biological processes and has been identified as a nucleotide exchange factor (GEF), a specific activator of the small GTPase Rac1 [11]; however, it is also specific for Cdc42 and to a lesser extent, RhoA [30]. The Par (comprising Par3, Par6, and atypical PKC)-Tiam1 complex stabilizes the front-rear polarization of migratory cells, thereby stimulating persistent and chemotactic migration. Meanwhile, in epithelial cells it controls the establishment of apical-basal polarity, thus showing the control of distinct forms of cellular polarity [12]. Tiam1-mediated Rac1 signaling is vital in establishing and maintaining of cell polarity, and it localizes the Rac1 activity at the leading edge of the migrating cells [8]. Furthermore, impaired Tiam1 signaling was reported to inhibit the formation of front-rear polarization during migration thereby inhibiting persistent migration [12].

The role of syndecan-4 transmembrane proteoglycan in directional migration, which is essential for several physiological processes, has been identified previously [21,25,31]. Moreover, syndecan-4, by regulating PKCα activity, is responsible for the localization of active Rac1 in membrane protrusions at the leading edge of cells [21], which is necessary for persistent migration. Therefore, knocking-out of syndecan-4 increases the level of delocalized active Rac1 [21]. It is noteworthy that the direct relationship between the syndecan-4 coordinated persistent migration and the Tiam1-mediated Rac1 activation remains unknown. Earlier, we reported that syndecan-4 binds and inhibits Tiam1, modulating the activity of Rac1 GTPase [24]. Thus, the syndecan-4-Tiam1 complex can regulate the local activity of Rac1. 

Both the total amount of Rac1-GTP and its spatial distribution is critical for cell migration. Pankov et al. reported that both very highly activated Rac1 and very low Rac1 activity inhibit migration [32]. Knocking-out of syndecan-4 results in increased Rac1-GTP level [21,25,26,27,28], thus syndecan-4 is involved to maintain the low basal activity of Rac1, and here we reported that syndecan-4 silencing decreased the migration of myoblasts. NSC23766 is a cell-permeable specific inhibitor of Rac1 activation by the Rac1-specific GEFs Tiam1 [29]. We hypothesized that Rac1 inhibitor treatment can improve the migratory parameters of syndecan-4 silenced cells, but the specific inhibition of Rac1 activity by NSC23766 did not rescue the effects of syndecan-4, rather exacerbated it. As NSC23766 treatment does not restore the migratory phenotype of the syndecan-4 knockdown cells, not only the active Rac1 level but the syndecan-4-dependent localization of Rac1 activity is also important for the correct cell migration. 

**Figure 5:**
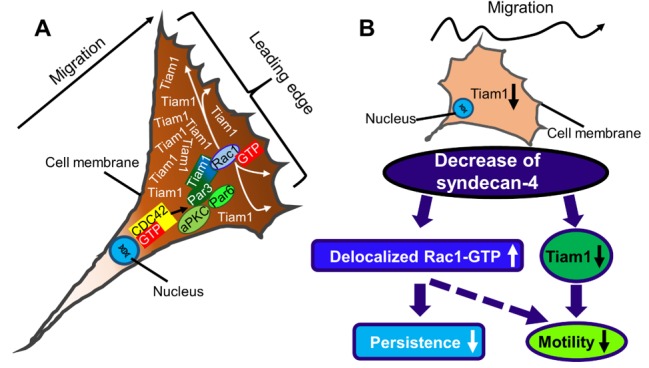
(**A**) Schematic representation of a migrating cell with a characteristic shape. The direction of migration is well defined, and the Tiam1 is asymmetrically accumulated at the leading edge resulting in Rac1 activation and lamellipodia formation. (**B**) Knocking-down of syndecan-4 resulted in decreased Tiam1 expression, abrogated the asymmetrical distribution of Tiam1, decreased the directional persistence of the movement, and decreased cell motility. Par3, Par6: Partitioning defective 3,6; aPKC: atypical PKC; Tiam1: T-lymphoma invasion and metastasis-1; 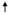: increase; 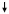: decrease.

For the effective cell migration, the establishment of a rear-front Rac1 gradient is essential. The syndecan-4 knockdown resulted in homogenous Tiam1 distribution, which might, in turn cause delocalized Rac1 activation (Figure 5). This can explain the reduced persistence and motility of syndecan-4 knockdown cells (Figure 5). In accordance with our observation, syndecan-4-null fibroblasts migrate randomly as a result of high delocalized Rac1 activity [21]. Interestingly, the polarized distribution of syndecan-4 was already shown during cytokinesis by its accumulation in the intercellular bridges during cell division [33]. Moreover, the directional persistence index of untreated syndecan-4 knockdown cells was similar to those of the NSC23766-treated non-transfected and scrambled lines; indicating that more than just the absolute amount of active Rac1 is important for the directionality of migration. 

In this study, Tiam1 exhibited a strong asymmetrical localization toward the leading edge in non-transfected and scrambled cell lines. The perinuclear accumulation of Tiam1 can also be observed indicating that Tiam1 localizes not only in the leading-edge protrusions but also shows significant accumulation in the Golgi-network in migrating cells. As with other cellular constituents, such as centrosomes, the Golgi-apparatus requires a leader-oriented localization for effective cell migration. The level of Tiam1 expression seems to be correlated with cell motility [34]. In our study, we detected a significantly higher signal intensity of Tiam1 in non-transfected and scrambled cell lines, than in the syndecan-4 silenced cells (Figure 4A). These findings may indicate increased expression of Tiam1 in control cell lines. Syndecan-4 silencing reduces Tiam1 signal intensity and abrogates the strong, asymmetrical Tiam1 gradient towards the leading edge. 

Given the role of Tiam1 and Rac1 in cell migration, several studies have explored the prognostic value of Tiam1 in patients with solid tumors. High Tiam1 expression has been significantly associated with shorter survival and positive lymphatic metastasis in patients with malignant solid tumors, and Tiam1 may present a promising prognostic biomarker and an effective therapeutic target for treating malignancies [35]. For example, a study has shown that Tiam1 expression correlated with cell motility in human breast cancer cells and is required to support the motile phenotype [34]. The authors reported the localization of endogenous Tiam1 to the Golgi, and demonstrated the role of this in Golgi reorientation, suggesting that it may support motility through a mechanism that is discrete from its known function in leading-edge dynamics [34]. Similarly, we identified accumulation of Tiam1 in intracellular compartments. In accordance with these Tiam1 expression data, the syndecan-4 knockdown cells in our model system exhibited both reduced expression of Tiam1 and reduced motility.

Overall, our results indicate a critical role of syndecan-4 during myoblast migration, which may contribute to an exploration of the essential role of syndecan-4 in the development and regeneration of skeletal muscle. We hope that our findings will facilitate understanding of the mechanisms underlying myoblast migration during embryonic development and postnatal muscle regeneration.

## 4. Materials and Methods 

### 4.1. Cell Culture and Plasmids

C2C12 mouse myoblast (ATCC; Manassas, VA, USA) cultures were maintained in medium containing 80% high-glucose Dulbecco’s modified Eagle’s medium (4.5 g/L glucose containing 584 mg/L glutamine and 110 mg/L pyruvate; Corning, New York, NY, USA), 20% fetal bovine serum (Gibco/Thermo Fisher Scientific, Waltham, MA, USA), and 65 µg/mL gentamicin (Lonza, Basel, Switzerland). Cells were transfected stably using shRNA (short hairpin RNA) expressing plasmids (OriGene, TR513122; Rockville, MD, USA) and X-tremeGENE transfection reagent (Roche, Basel, Switzerland). For syndecan-4 knockdown, we applied plasmids targeting the following sequences: 5′-GAA CTG GAA GAG AAT GAG GTC ATT CCT AA-3′ (shSDC4#1) or 5′-GCG GCG TGG TAG GCA TCC TCT TTG CCG TT-3′ (shSDC4#2). The scrambled plasmid targeted the 5′-GCA CTA CCA GAG CTA ACT CAG ATA GTA CT-3′ sequence. Selection of the transfected cells was obtained by adding 4 μg/mL puromycin (Sigma-Aldrich, St. Louis, MO, USA) to the culturing medium.

### 4.2. Time-Lapse Imaging of Live Cells

Live-cell imaging was carried out using the Operetta high-content imaging system with a 20× objective (PerkinElmer, Inc., Waltham, MA, USA). For imaging, cells were seeded in 24-well plates at a density of 7.5 × 10^3^ cells/well. After 60 min, the medium was changed to a serum-reduced one to suppress cell division, and 24 h later the nuclei were stained with Hoechst33342 (1:2000, 1 mg/mL stock solution; Sigma-Aldrich), and Rac1 was inhibited by NSC23766 treatment during the measurement (50 µM; Tocris Bioscience, Bristol, United Kingdom). Time-lapse images were obtained automatically at 20 min intervals for 18 h at 37 °C and 5% CO_2_.

### 4.3. Single-Cell Tracking of Cultured Myoblasts

Time-lapse microscopic images were analyzed using ImageJ (National Institutes of Health, Bethesda, MD, USA, https://imagej.nih.gov/ij/) and CellTracker (http://celltracker.website/) software programs. The nuclei were tracked manually through every frame, and the x and y coordinates of the movement were recorded. We excluded dying, dividing, or damaged cells from the analysis. Based on the nuclear coordinates, the length of total path, the vectorial distance (i.e., real shift of the cell), and maximal distance from origin, the average and maximal speed were calculated. The directionality of the cell movement was described by the persistence index, which was defined by the ratio of the vectorial distance (the distance between the origin and the endpoint of the movement) and the length of total path.

### 4.4. Trajectories and Wind-Rose Diagrams

To illustrate the trajectories, the migratory tracks of the individual cells were transposed to a common origin using Excel DiPer Plot_At_Origin macro [36]. The wind rose plots created draw individual cell paths based on the *x* and *y* coordinates of the cell tracking data.

### 4.5. Fluorescent Staining and Microscopy

For fluorescent staining, cells were seeded for 24 h onto fetal bovine serum (Gibco/Thermo Fisher Scientific, Waltham, MA, USA) coated glass coverslips (Hirschmann Laborgeräte GmbH & Co. KG, Eberstadt, Germany), fixed with 4% paraformaldehyde (Molar Chemicals Kft., Halásztelek, Hungary) for 10 min at room temperature, permeabilized with 0.3% Tween 20 (Sigma-Aldrich, Inc., St. Louis, Missouri, USA), and blocked with 1% bovine serum albumin (Sigma-Aldrich) in PBS. Rabbit polyclonal anti-Tiam1 primary antibody (OST00085W; Invitrogen, Carlsbad, CA, USA) was visualized with the appropriate Alexa488-conjugated secondary antibody (Invitrogen). The nuclei were counterstained with Hoechst 33258 (0.01 mg/mL, Sigma-Aldrich). Epi-fluorescence images were obtained on a Nikon Eclipse Ti-E microscope (Nikon Instruments Inc., 1300 Walt Whitman Road Melville, NY 11747-3064, USA) with 40× objectives.

### 4.6. Evaluation of Tiam1 Immunostaining

The epifluorescent images were recolored as heat maps using ImageJ (National Institutes of Health, Bethesda, MD, USA, https://imagej.nih.gov/ij/) image analysis program (Image>Lookup Tables). Each pixel was colored based on the pixel intensity value (16-bit images; 0-65535 gray value) according to the scale bar shown in the images (the lowest intensity pixels are shown as blue; the highest intensity pixels are shown as red). The contours of the individual cells were drawn (Analyze>Analyze particles), and the average pixel intensity within the border of the cells were quantified (Analyze>Measure) in the different cell lines. The intensity value of each pixel was measured within the selected area and the sum of the intensities was divided by the area of the cell to obtain the average Tiam1 intensity value of the individual cells. To measure the amount of variation in the pixel intensities, the standard deviation of the intensity values was also calculated in every cell (Analyze>Measure).

### 4.7. Statistical Analysis

Differences between groups were analyzed using a one-way ANOVA, followed by the Scheffe post-hoc test. GraphPad Prism 7.0 (GraphPad Software Inc., San Diego, CA, USA) was used for graphing and statistical analyses. The data are expressed as means + standard errors of the means. A *p* value < 0.05 was considered as significantly different.

## Supplementary Materials

Supplementary materials can be found at https://www.mdpi.com/1422-0067/21/3/823/s1.

## Abbreviations

GDI	Guanine dissociation inhibitors
GEF	Guanine nucleotide exchange factor
GAP	GTPase activating protein
Par	Partitioning defective
PKCα	Protein kinase C α
Rac1	Ras-related C3 botulinum toxin substrate 1
SDC4	Syndecan-4
Tiam1	T-lymphoma invasion and metastasis-1
